# Engineering of Insulin Receptor Isoform-Selective Insulin Analogues

**DOI:** 10.1371/journal.pone.0020288

**Published:** 2011-05-20

**Authors:** Tine Glendorf, Carsten E. Stidsen, Mathias Norrman, Erica Nishimura, Anders R. Sørensen, Thomas Kjeldsen

**Affiliations:** Diabetes Research Unit, Novo Nordisk A/S, Maaloev, Denmark; Boston University, United States of America

## Abstract

**Background:**

The insulin receptor (IR) exists in two isoforms, A and B, and the isoform expression pattern is tissue-specific. The C-terminus of the insulin B chain is important for receptor binding and has been shown to contact the IR just adjacent to the region where the A and B isoforms differ. The aim of this study was to investigate the importance of the C-terminus of the B chain in IR isoform binding in order to explore the possibility of engineering tissue-specific/liver-specific insulin analogues.

**Methodology/Principal Findings:**

Insulin analogue libraries were constructed by total amino acid scanning mutagenesis. The relative binding affinities for the A and B isoform of the IR were determined by competition assays using scintillation proximity assay technology. Structural information was obtained by X-ray crystallography. Introduction of B25A or B25N mutations resulted in analogues with a 2-fold preference for the B compared to the A isoform, whereas the opposite was observed with a B25Y substitution. An acidic amino acid residue at position B27 caused an additional 2-fold selective increase in affinity for the receptor B isoform for analogues bearing a B25N mutation. Furthermore, the combination of B25H with either B27D or B27E also resulted in B isoform-preferential analogues (2-fold preference) even though the corresponding single mutation analogues displayed no differences in relative isoform binding affinity.

**Conclusions/Significance:**

We have discovered a new class of IR isoform-selective insulin analogues with 2–4-fold differences in relative binding affinities for either the A or the B isoform of the IR compared to human insulin. Our results demonstrate that a mutation at position B25 alone or in combination with a mutation at position B27 in the insulin molecule confers IR isoform selectivity. Isoform-preferential analogues may provide new opportunities for developing insulin analogues with improved clinical benefits.

## Introduction

Insulin is a small globular protein composed of two polypeptide chains, A (21 residues) and B chain (30 residues), which are covalently linked by two interchain disulfide bridges [1]. The biological effects of insulin are mediated through the insulin receptor (IR), a covalent dimer consisting of two extracellular insulin-binding α-subunits and two transmembrane β-subunits disulfide-bonded in a β-α-α-β conformation (reviewed in [2]). Due to alternative splicing of exon 11 of the IR gene, the IR exists as two isoforms, isoform A (IR-A) and isoform B (IR-B), which differ in the absence or presence of a 12 amino acid sequence, respectively, located at the C-terminal end of the α-subunit [3]. The isoform expression pattern is regulated in a highly tissue-specific manner, which is more or less conserved amongst species [3]–[6]. In humans, both isoforms are present in tissues such as skeletal muscle, adipose tissue, kidney, placenta, and heart; however, IR-B is the most abundant IR isoform found in the insulin-responsive tissues. The IR-B is predominantly expressed in the liver (>90–95% IR-B) and this expression pattern appears highly conserved among species, while IR-A is the dominant isoform found in the brain, spleen, fetal tissues, as well as in several cancers [3]–[10]. The abundance of the two isoforms has further been suggested to be dependent on cell differentiation and developmental stage [6], [7]. The two insulin receptor isoforms have also been reported to display functional differences such as slight differences in insulin affinity, internalization kinetics as well as signalling capacity and dynamics [11]–[15], but one of the most distinct being the difference in IGF-I and IGF-II affinity [7], [16]. Compared to insulin binding, both IR isoforms display low IGF-I binding affinities, however, IR-A has a higher affinity for both IGFs than IR-B and is in fact able to bind IGF-II with an affinity close to that of human insulin (5-fold lower affinity). Nonetheless, the exact physiological significance of the above-mentioned observations remains unclear and research regarding the functional differences between the two IR isoforms is therefore still ongoing. Decades of investigations on insulin-receptor interactions have generated numerous insulin analogues with different IR binding properties. However, in this paper, we describe for the first time the engineering of receptor isoform-selective insulin analogues.

The present study was initially motivated by the multifaceted role of the C-terminal end of the B chain. This segment of the insulin molecule plays a fundamental role in dimer formation [1], [17], which is important for insulin storage in the β-cell. In addition, the B chain C-terminus is of great importance for receptor binding [18]–[21]. Residues B23–B26 are considered part of insulin's ‘classical’ binding surface [18] and are also essential for the negative cooperativity observed in binding [22]. Photo-affinity cross-linking studies have in fact established a direct contact between B24–B27 derivatives and distinct sites on the IR [21], [23]–[25]. Another interesting finding is that deletion of residues B26–B30 does not affect the *in vitro* binding potency of insulin given that PheB25 is amidated [26]; however, studies of modifications in the C-terminus of the B chain have provided insulin analogues with interesting binding properties [19], [20], [27]. Furthermore, it is a commonly held view that the B chain C-terminus detaches from the hydrophobic core and rearranges to expose a buried functional surface comprising PheB24 and part of the N-terminal A chain upon receptor binding [28]–[31], although the extent of the conformational change remains unknown. Interestingly, position B24 and B25 are also sites of diabetes-associated mutations (B24Ser – insulin *Los Angeles*; B25Leu – insulin *Chicago*) [32], which underline the importance of the aromatic region in receptor binding.

We therefore decided to perform a comprehensive structure-function analysis on certain positions in the C-terminal part of the insulin B chain by total amino acid scanning mutagenesis, a powerful method recently developed [33] by which the resulting single-substitution insulin analogues are individually evaluated in terms of relative IR-A and IR-B binding potencies compared to human insulin. The initial library screening and the following engineering of library combinations resulted in the discovery of analogues with 2–4-fold differences in relative binding affinities for the two receptor isoforms.

## Materials and Methods

### Miscellaneous

Human insulin (HI), [^125^I]TyrA14-labelled insulin and immobilized *Achromobacter lyticus* protease (ALP) were from Novo Nordisk A/S. Binding assays were performed using IR-specific monoclonal mouse antibody 83-7 [34] licensed from Dr. K. Siddle (University of Cambridge, Cambridge, U.K.) and solubilized human insulin receptor (holoreceptor) semipurified by wheat germ agglutinin chromatography from BHK cells, which were stably transfected with the pZem vector containing either the human IR-A or IR-B insert [35]. Materials, strains, general molecular biology techniques, purification of analogues and receptors, and yeast expression system were as previously published ([33] and references therein) unless otherwise mentioned.

### Analogue construction and expression in yeast

Vector construction, precursor expression and conversion, and quantification of the insulin analogues were performed as recently published [33]. Briefly, insulin precursor DNA constructs were transformed into *Saccharomyces cerevisiae* strain MT663. The insulin precursors were expressed as proinsulin-like fusion proteins (5 mL cultures), with an N-terminal removable spacer peptide and mini C-peptide. The single-chain precursors were enzymatically converted into mature two-chain desB30 (insulin lacking the amino acid at position B30) insulin analogues using lysine specific ALP. Analogue concentrations were determined by reverse-phase high-performance liquid chromatography using human insulin as an external standard. All amino acid substitutions and full conversion of the precursors were confirmed by matrix-assisted laser desorption ionization time-of-flight mass spectrometry.

### Insulin receptor binding assay

IR binding of the insulin analogues were determined by competition of [^125^I]TyrA14-labelled insulin binding in a scintillation proximity assay (SPA) (analogues containing Cys and Lys substitutions were excluded from the SPA due to potential disulfide misparring or probable ALP cleavage in the conversion step, respectively) as recently published [33] and data from the SPA were analyzed according to the four-parameter logistic model [36] assuming a common slope, basal and maximal response level of the curves for human insulin and the insulin analogues. The percentage of tracer bound in the absence of competing ligand was less than 15% in all assays, to prevent ligand-depletion artifacts and ∼14-fold changes in responses were obtained. The affinities (picomolar affinity range) of the analogues are calculated relative to that of human insulin [IC_50(insulin)_/IC_50(analogue)_×100%] measured within the same plate.

### Crystal structure determination

Crystals were obtained by the sitting drop vapor diffusion method from a reservoir solution containing 0.8 M K/NaTartrate, 0.1 M Tris pH 8.5, 0.5% PEG MME 5000 and belong to the cubic space group (I2_1_3). Data were collected with a rotating anode (Rigaku, MicroMax-007HF) equipped with a MarCCD detector and processed by XDS [37]. The structure was solved by molecular replacement using Molrep [38] with an in house structure as search model. Data refinement and model building was made using the programs Refmac [39] and Coot [40]. Further details about data collection and refinement statistics are available as Supporting Information (Table S1) on the PLoS ONE web site.

## Results and Discussion

Insulin analogues with 2-fold differences in relative IR isoform affinity were discovered by amino acid scanning mutagenesis of the aromatic locus PheB24-PheB25-TyrB26 and the two neighbouring positions in the C-terminus of the B chain. Subsequently, the concept of differential IR isoform binding was further examined by engineering several analogues with multiple mutations of which some were identified as having 2–4-fold differences in relative IR isoform affinity. Both IR-A and IR-B-selective analogues were engineered in the present study and position B25 was identified as the common denominator involved in receptor isoform selectivity.

**Figure 1:**
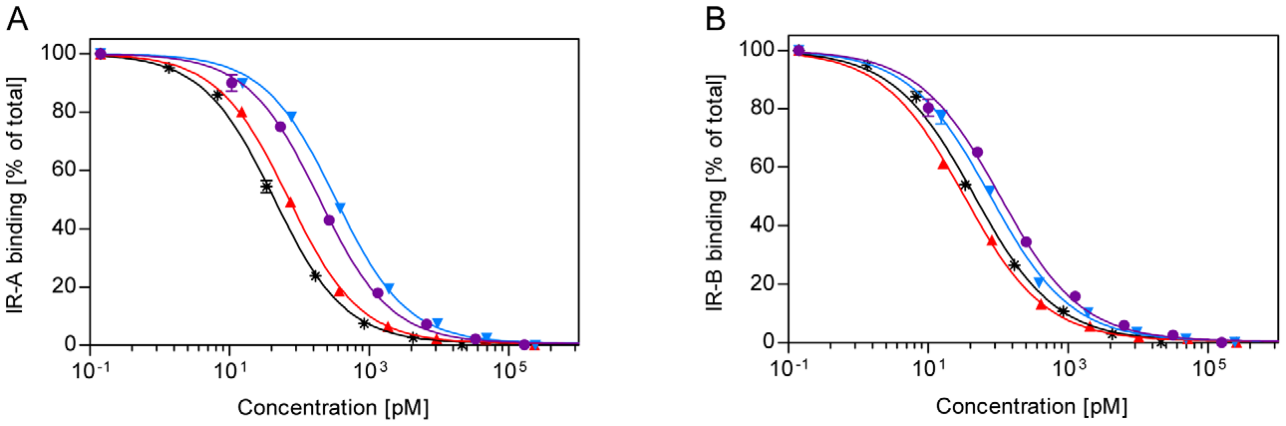
Representative IR competition binding curves. Displacement of ^125^I-labelled insulin by human insulin and insulin analogues from the (**A**) A-isoform or the (**B**) B-isoform of the IR. The amount of bound ^125^I-labelled insulin as a percentage of bound ^125^I-labelled insulin in the absence of unlabelled analogue is plotted against the concentration of unlabelled analogue. Data points are means ± SEM of four measurements within one assay (n = 1). The ^125^I-labelled insulin was displaced with purified human insulin standard (*) or insulin analogues [A8H, B25H, B27E] (▴), [A8H, B25N, B27E] (▾), and [A8H, B25N] (•).

The insulin precursors were individually expressed in *S. cerevisiae* and the analogue precursor expression yields of the corresponding insulin analogues (analogues listed in Table 1), all exceeded that of wild-type precursor, indicating correct processing and folding in the yeast cell [41]. Following enzymatic conversion of the precursors into mature two-chain insulin analogues, the relative receptor binding affinities of the analogues were determined using either IR-A or IR-B (wild-type holoreceptor) purified from BHK cells (see [33] for a more detailed description of the scintillation proximity assay setup). The difference in IR isoform binding was confirmed by a membrane-bound receptor assay for a selection of the analogues (data not shown). The validity of using insulin analogues taken directly from yeast supernatant in the IR binding experiments has previously been clearly established [33]. Examples of competition binding curves are given in Figure 1.

**Table 1:** Relative insulin receptor binding affinities of the analogues.

Mutations	IR-A affinity[% of human insulin]	IR-B affinity[% of human insulin]	IR-B/IR-A
None	98±4	100±5	1
A8H	308	308	1
B25A	3±0.3	6±0.8	2
B25H	33	32	1
B25Y	285±78	157±27	0.6
B27D	74	73	1
B27E	83	93	1
B25N	4±0.2	8±0.7	2
A8H, B25N	22±4	44±5	2
B25H, B27E	13±1	30±5	2
A8H, B25A, B27E	10±1	24±2	2
A8H, B25Y, B27E	714±31	459±51	0.6
A8H, B25N, B27D	10±1	38±4	4
A8H, B25N, B27E	15±3	57±3	4
A8H, B25H, B27D	43±4	82±7	2
A8H, B25H, B27E	67±3	140±5	2

For analogues tested in at least three independent experiments, the mean ± SD is presented. Analogues with no fold difference between IR-A and IR-B binding affinity were tested once as part of the analogue library screening.

It has been reported that human insulin exhibits a ∼2-fold higher absolute affinity for IR-A than for IR-B, though both receptor isoforms bind insulin with high affinity [5], [11] and it is therefore important to note that in the present study the relative binding affinities of the analogues were compared to human insulin and solely by comparing the human insulin standard and insulin analogue tested within the same plate. When performing binding assays on both IR-A and IR-B a very high correlation between the relative affinities determined for the two receptor isoforms is normally observed (see ref [33], which includes >100 insulin analogues). We routinely perform IR assays on vast numbers of insulin analogues including the commercially available insulin analogues and X10 (also known as AspB10) on both isoforms of the receptor. We do not find that any of these analogues display isoform selectivity (manuscript in preparation), including insulin detemir and insulin aspart. It has recently been reported that insulin X10 displays a 3-fold higher relative affinity for IR-A than IR-B compared to human insulin [42]; however this is in contrast to our findings (manuscript in preparation) and to Sciacca et al. [43]. Furthermore, we have tested the desB30 version of X10 [B10D, desB30] as well as [B10E, desB30] and determined that these analogues bind with the same relative affinity to both isoforms of the IR [33], which is in accordance with our X10 data.

In this study, single-mutation libraries were constructed and evaluated in terms of receptor binding potency for both isoforms of the IR. Interestingly, anomalies between the relative IR-A and IR-B binding affinities were found in the [B25X] library, in which X represents any of the 20 naturally occurring amino acids. The systematic amino acid scanning mutagenesis revealed that analogues [B25A] and [B25N] had increased preferences for IR-B compared to IR-A with respect to binding, while the opposite was observed for the [B25Y] analogue (see Table 1). As expected, the vast majority of the amino acid substitutions at position B25 disrupted receptor binding, being markedly decreased for both the [B25A] and [B25N] analogues, whereas an increase in affinity was observed for the [B25Y] analogue. The IR-B preferential [B25N] analogue, which displayed a 2-fold higher relative binding affinity for IR-B (8±0.7%) compared to IR-A (4±0.2%), was further investigated. Due to the location of a Thr residue at B27, the replacement of PheB25 with Asn creates a potential glycosylation site and analysis by RP-HPLC and LC-MS showed that approximately 35% of the secreted B25N precursor was glycosylated. In the above mentioned binding affinities for the [B25N] analogue, the glycosylated [B25N] analogue is assumed not to participate in IR binding.

To further validate the observed discrepancy in receptor isoform binding for this analogue, an A8H mutation was combined with the B25N mutation in order to increase the IR binding affinity of the analogue and thereby also the robustness of the assay. The A8H mutation has been demonstrated to cause an approximately 3-fold increase in both IR binding and biological activity compared to human insulin [44] and the binding data from the [A8H] analogue in this study confirms the 3-fold increase in binding affinity for both isoforms of the IR (see Table 1). For the majority of protein-protein interactions, near simple additivity applies for the free energy changes derived from multiple mutations [45]. This is also generally observed for human insulin where the additivity of the mutational effects often is employed when designing analogues. However, exceptions can occur if the mutated residues interact with each other or produce large structural perturbations. Here, the A8H mutation is not in close proximity to the aromatic patch at the C-terminal end of the B chain and the mutation is therefore not believed to cause any long-range structural perturbations, which can affect the divergence in receptor isoform affinity [46].

Approximately 20% of the resulting [A8H, B25N] analogues were found to be glycosylated, but more importantly the 2-fold difference in relative IR isoform binding affinity was preserved. The analogue displayed relative binding potencies of 22±5% and 44±5% for IR-A and for IR-B, respectively, compared to human insulin, when the glycosylated analogue was assumed not to participate in IR binding. To ensure that glycosylation had no impact on the observed divergence in relative IR isoform affinity, the yeast strain expressing [A8H, B25N] was fermented and the secreted precursor enzymatically cleaved and purified (98% purity). The purified and unglycosylated [A8H, B25N] analogue retained its 2-fold difference in relative IR binding potency demonstrating that glycosylation had no influence on the difference in relative IR isoform binding.

Whereas PheB24 and TyrB26 pack against the core of the molecule [1], the side chain at position B25 projects from the surface of insulin and is able to form contacts with the amino acid side chain at position B27, which also protrudes outwards from the molecule. The [A8H, B25N, B27X] library was therefore constructed in order to examine whether amino acid substitutions of ThrB27 would affect the divergence in relative IR isoform affinity observed for the [A8H, B25N] analogue. Interestingly, the analogues from the [A8H, B25N, B27X] library all displayed ≥2-fold differences (IR-B>IR-A) in relative binding affinities for the two receptor isoforms. In fact, analogues [A8H, B25N, B27D] and [A8H, B25N, B27E] both exhibited a 4-fold higher relative binding affinities for IR-B than IR-A (see Table 1). However, the single mutation analogues [B27D] and [B27E] showed no considerable difference in relative receptor isoform binding suggesting that in this analogue library, B25N is the determinant of IR-B selectivity, while an acidic amino acid residue in position B27 further enhances the difference in isoform binding.

To evaluate whether the remaining possible amino acid substitutions at B25 including the IR-A preferential Tyr and IR-B preferential Ala substitutions would be affected by an acidic amino acid residue at position B27 with respect to IR isoform binding, the [A8H, B25X, B27E] library was constructed and receptor binding evaluated. The triple mutation library confirmed the observed 2-fold differences in relative receptor isoform affinity and the [A8H, B25A, B27E] analogue displayed relative binding affinities of 10±1% for IR-A and 24±2% for IR-B, while the [A8H, B25Y, B27E] analogue showed highly increased binding affinities of 714±31% and 459±51% for IR-A and IR-B, respectively, compared to human insulin. Surprisingly, the [A8H, B25H, B27E] analogue was found to exhibit IR-B selectivity (67±3% on IR-A and 140±5% on IR-B). The result was considered puzzling given that either the [B25H] or [B27E] analogue showed no considerable difference in relative IR isoform binding affinity. To substantiate these results, the [B25H, B27E] and [A8H, B25H, B27D] analogues were constructed and receptor binding evaluated (see Table 1). Both analogues showed 2-fold higher relative IR-B binding compared to IR-A and confirmed that a His mutation at position B25 in combination with an acidic amino acid at position B27 results in IR-B selectivity of the analogue.

When searching for an isoform selective insulin analogue, it seemed plausible that a mutation in the C-terminus of the B chain would be essential for isoform selectivity given that B25 and B27 have been shown to cross-link to a region at the C-terminus of the IR α-chain, a segment known as the CT peptide, just adjacent to the 12-amino acid segment encoded by exon 11 [21], [23], [24]. The CT peptide has been suggested to be directly involved in insulin binding [21], [47]–[49] and alanine mutations in this region have also been shown to have differential effects on the ligand affinity of the two receptor isoforms [50]. The fact that certain IR alanine mutations are isoform-specific as shown by Whittaker et al. [50] indicates that the structures of the IR-A and IR-B binding epitopes differ and our findings corroborate this concept. This is also supported by the difference in IR isoform affinity observed for IGF-I and IGF-II [7], [16]. Insulin and the IGFs have overlapping receptor binding sites [51]; however, IGF-II is able to bind to IR-A, but not to IR-B with an affinity similar to that of insulin [7], [16].

While it is clear that position B25 is a key determinant for isoform selectivity, the structure-activity relationships are less obvious. A comparison of the relative binding affinities of the isoform-specific analogues reveals that simple additivity of the mutational effects does not apply for the analogues with mutations both at position B25 and B27. This indicates an interaction between the substituted residues. Analogue [B25N] displays the same fold increase for both receptor isoforms when combined with the A8H mutation, whereas the addition of B27E or B27D to the A8H and B25N substitution causes a disproportionate change in relative IR isoform affinities of the resulting analogues. The addition of B27E or B27D would be expected to result in a decrease in receptor affinity of the triple mutated analogues assuming no intramolecular interactions occur and simple additivity of the free energy change of the single mutations applies. However, the expected decreases in relative binding affinities were only evident for the A isoform, whereas increases in relative binding affinities were observed for the B isoform of the receptor (see Table 1).

**Figure 2:**
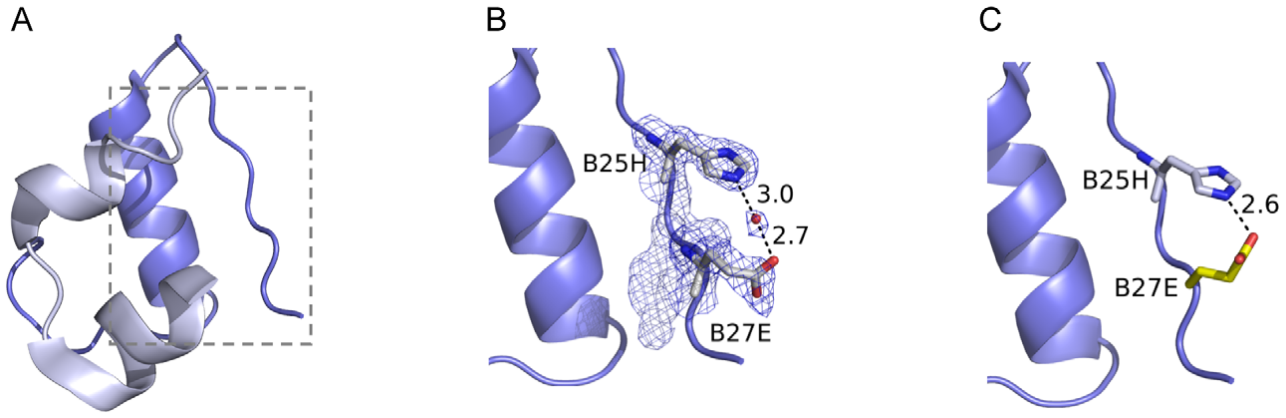
Structure of the [B25H, B27E] insulin analogue. (**A**) Overview of the [B25H, B27E] insulin analogue with the A chain and B chain depicted in light- and dark blue, respectively. The segment shown in panel B and C is indicated by a gray dotted line in A. In (**B**), the 2Fo-Fc electron density is shown for residues B25H to B27E contoured at 1 σ. A hydrogen bond interaction is mediated by a water molecule (red sphere), but no direct interaction is shown between B25H and B27E. In (**C**), a modelled B27E residue is shown (yellow) in another rotamer orientation to indicate that a direct interaction between B25H and B27E could be possible. Distances are shown in Ångstrom.

To gain a further understanding of the structure-activity relationships for the B isoform-specific mutations, crystal structures of analogues [A8H, B25N], [A8H, B25N, B27E] and [A8H, B25H, B27E] were obtained by X-ray crystallography. As expected, the A8H mutation did not cause any long-range structural perturbations that affected the C-terminus of the B chain, but under the conditions for crystal growth, no intramolecular interactions between the B25 and B27 side chains could be observed (see Figure 2B). However, the potential effect of crystal packing forces has to be taken into account, when investigating the intramolecular interactions between residues located in the C-terminal end of the B-chain, which is part of the insulin dimer interface. Dimer forming interactions could be preferred prior to any possible B25–B27 interactions. Furthermore, the pH of the crystallisation buffer was 8.5. At this pH the side chain of B25H is most likely unprotonated. The binding assays were performed at near physiological pH, where B25H is more likely to be protonated and would thus attract the unprotonated B27E. A modelled structure was prepared, where the side chain rotamer of B27E was varied to see whether the distance between B25H and B27E would permit an interaction. Figure 2C shows that the distance between the two residues is 2.6 Å, which suggests that a hydrogen bond interaction would be possible. Complete clarification of the molecular details for isoform-selectivity will most likely require a crystal structure of both receptor isoforms in complex with insulin and/or the isoform-specific analogues. Solution structures of the analogues by NMR spectroscopy may also provide structural insight on isoform selectivity.

In conclusion, total amino acid scanning mutagenesis of positions B23–B27 of insulin and the further engineering of a selection of multi-substituted analogues allowed for the construction of both IR-A and IR-B isoform-specific insulin analogues and to identify position B25 as a key determinant for isoform selectivity. The selectivity is reflected by a larger decrease in affinity for one isoform than the other. Our results demonstrated that a replacement of PheB25 with either Ala or Asn resulted in an analogue with a 2-fold higher relative binding affinity for the B isoform compared to the A isoform of the receptor, whereas the opposite was observed when introducing a Tyr residue at this position. Combining the A8H and B25N mutations with an acidic amino acid residue at position B27 caused an additional 2-fold selective increase in affinity for the B isoform of the receptor resulting in analogues displaying 4-fold IR isoform preferences with respect to binding. Surprisingly, the combination of B25H with either B27E or B27D was also found to result in IR-B preferential analogues (2-fold differences) despite the fact that the corresponding single mutation analogues displayed no differences in relative isoform binding affinity.

Endogenously produced insulin is secreted from the pancreas directly into the portal vein and the liver is therefore exposed to much higher concentrations of insulin than the periphery [52], whereas subcutaneous administration of exogenous insulin leads to a non-physiological insulin distribution, in which peripheral tissues such as muscle and fat become relatively ‘over-insulinized’. Since the tissue-specific isoform expression pattern is highly regulated and IR-B is the vastly abundant isoform expressed in the liver [3]–[5], an IR-B selective insulin analogue may demonstrate liver-selectivity and consequently mimic the natural route of insulin distribution. This could lead to improved blood glucose control with a reduced risk of hypoglycaemia. In addition, a liver-selective analogue may also lead to improved lipid profiles and a reduced fat mass compared to the currently available insulin therapies.

To summarize, we have engineered several different insulin receptor isoform-specific insulin analogues. Both IR-A and IR-B selective analogues were constructed and these findings represent the first description of receptor isoform-selective insulin analogues. A more detailed characterization of the isoform-selective analogues and their biological effects is requisite in order to fully understand the underlying basis for this phenomenon and its biological consequences (further *in vivo* and *in vitro* data are pending submission). However, the insulin analogues presented in this study may open new avenues for the engineering of a new class of tissue-specific/liver-selective insulin analogues with improved clinical benefits.

## Supporting Information

Table S1 Data collection and definement statistics.
(DOC)

## References

[1] Baker EN, Blundell TL, Cutfield JF, Cutfield SM, Dodson EJ (1988). The structure of 2Zn pig insulin crystals at 1.5 Å resolution.. Philos Trans R Soc Lond B Biol Sci.

[2] Ward CW, Lawrence MC (2009). Ligand-induced activation of the insulin receptor: a multi-step process involving structural changes in both the ligand and the receptor.. BioEssays.

[3] Seino S, Bell GI (1989). Alternative splicing of human insulin receptor messenger RNA.. Biochem Bioph Res Co.

[4] Moller DE, Yokota A, Caro JF, Flier JS (1989). Tissue-specific expression of two alternatively spliced insulin receptor mRNAs in man.. Mol Endocrinol.

[5] Mosthaf L, Grako K, Dull TJ, Coussens L, Ullrich A (1990). Functionally distinct insulin receptors generated by tissue-specific alternative splicing.. EMBO J.

[6] Serrano R, Villar M, Martinez C, Carrascosa JM, Gallardo N (2005). Differential gene expression of insulin receptor isoforms A and B and insulin receptor substrates 1, 2 and 3 in rat tissues: modulation by aging and differentiation in rat adipose tissue.. J Mol Endocrinol.

[7] Frasca F, Pandini C, Scalia P, Sciacca L, Mineo R (1999). Insulin receptor isoform A, a newly recognized, high-affinity insulin-like growth factor II receptor in fetal and cancer cells.. Mol Cell Biol.

[8] Vella V, Pandini G, Sciacca L, Mineo R, Vigneri R (2002). A novel autocrine loop involving IGF-II and the insulin receptor isoform-A stimulates growth of thyroid cancer.. J Clin Endocr Metab.

[9] Kalli KR, Falowo OI, Bale LK, Zschunke MA, Roche PC (2002). Functional insulin receptors on human epithelial ovarian carcinoma cells: implications for IGF-II mitogenic signaling.. Endocrinology.

[10] Sciacca L, Mineo R, Pandini G, Murabito A, Vigneri R (2002). In IGF-I receptor-deficient leiomyosarcoma cells autocrine IGF-II induces cell invasion and protection from apoptosis via the insulin receptor isoform A.. Oncogene.

[11] Yamaguchi Y, Flier JS, Yokota A, Benecke H, Backer JM (1991). Functional properties of two naturally occurring isoforms of the human insulin receptor in Chinese hamster ovary cells.. Endocrinology.

[12] Kellerer M, Lammers R, Ermel B, Tippmer S, Vogt B (1992). Distinct α-subunit structures of human insulin receptor A and receptor B variants determine differences in tyrosine kinase activities.. Biochemistry.

[13] Kosaki A, Pillay TS, Xu L, Webster NJG (1995). The B isoform of the insulin receptor signals more efficiently than the A isoform in HepG2 cells.. J Biol Chem.

[14] McClain DA (1991). Different ligand affinities of the two human insulin receptor splice variants are reflected in parallel changes in sensitivity for insulin action.. Mol Endocrinol.

[15] Giudice J, Leskow FC, Arndt-Jovin DJ, Jovin TM, Jares-Erijman EA (2011). Differential endocytosis and signaling dynamics of insulin receptor variants IR-A and IR-B.. J Cell Sci.

[16] Benyoucef S, Surinya KH, Hadaschik D, Siddle K (2007). Characterization of insulin/IGF hybrid receptors: contributions of the insulin receptor L2 and Fn1 domains and the alternatively spliced exon 11 sequence to ligand binding and receptor activation.. Biochem J.

[17] Blundell TL, Dodson G, Hodgkin D, Mercola D (1972). The structure in the crystal and its reflection in chemistry and biology.. Adv Protein Chem.

[18] Pullen RA, Lindsay DG, Wood SP, Tickle IJ, Blundell TL (1976). Receptor-binding region of insulin.. Nature.

[19] Nakagawa SH, Tager HS (1987). Role of the COOH-terminal B-chain domain in insulin receptor interactions.. J Biol Chem.

[20] Mirmira RG, Nakagawa SH, Tager HS (1991). Importance of the character and configuration of residues B24, B25, and B26 in insulin-receptor interactions.. J Biol Chem.

[21] Xu B, Huang K, Chu YC, Hu SQ, Nakagawa S (2009). Decoding the cryptic active conformation of a protein by synthetic photoscanning. Insulin inserts a detachable arm between receptor domains.. J Biol Chem.

[22] De Meyts P, Van Obberghen E, Roth J, Wollmer A, Brandenburg D (1978). Mapping of residues responsible for negative cooperativity of receptor-binding region of insulin.. Nature.

[23] Xu B, Hu SQ, Chu YC, Huang K, Nakagawa SH (2004). Diabetes-associated mutations in insulin: consecutive residues in the B chain contact distinct domains of the insulin receptor.. Biochemistry.

[24] Kurose T, Pashmforoush M, Yoshimasa Y, Carroll R, Schwartz GP (1994). Cross-linking of a B25 azidophenylalanine insulin derivative to the carboxyl-terminal region of the α-subunit of the insulin receptor. Identification of a new insulin-binding domain in the insulin receptor.. J Biol Chem.

[25] Shoelson SE, Lee JS, Lynch CS, Backer JM, Pilch PF (1993). Bpa^B25^ insulins. Photoactivatable analogues that quantitatively cross-link, radiolabel, and activate the insulin receptor.. J Biol Chem.

[26] Fischer WH, Saunders D, Brandenburg D, Wollmer A, Zahn H (1985). A ahortened insulin with full *in vitro* potency.. Biol Chem H-S.

[27] Zakova L, Kazdova L, Hanclova I, Protivinska E, Sanda M (2008). Insulin analogues with modifications at position B26. Divergence of binding affinity and biological activity.. Biochemistry.

[28] Hua QX, Shoelson SE, Kochoyan M, Weiss MA (1991). Receptor binding redefined by a structural switch in a mutant human insulin.. Nature.

[29] Ludvigsen S, Olsen HB, Kaarsholm NC (1998). A structural switch in a mutant insulin exposes key residues for receptor binding.. J Mol Biol.

[30] Nakagawa SH, Tager HS (1992). Importance of aliphatic side chain structure at position 2 and 3 of the insulin A chain in insulin-receptor interactions.. Biochemistry.

[31] Markussen J, Jørgensen KH, Sørensen AR, Thim L (1985). Single chain des-(B30) insulin. Intramolecular crosslinking of insulin by trypsin-catalyzed transpeptidation.. Int J Pept Prot Res.

[32] Shoelson S, Haneda P, Blix P, Nanjo A, Sanke T (1983). Three mutant insulins in man.. Nature.

[33] Glendorf T, Sørensen AR, Nishimura E, Pettersson I, Kjeldsen T (2008). Importance of the solvent-exposed residues of the insulin B chain α-helix for receptor binding.. Biochemistry.

[34] Soos MA, Siddle K, Baron MD, Heward JM, Luzio JP (1986). Monoclonal antibodies reacting with multiple epitopes on the human insulin receptor.. Biochem J.

[35] Slaaby R, Schäffer L, Lautrup-Larsen I, Andersen AS, Shaw AC (2006). Hybrid receptors formed by insulin receptor (IR) and insulin-like growth factor I receptor (IGF-IR) have low insulin and high IGF-1 affinity irrespective of the IR splice variant.. J Biol Chem.

[36] Vølund A (1978). Application of four-parameter logistic model to bioassay: comparison with slope ratio and parallel line models.. Biometrics.

[37] Kabsch W (1993). Automatic processing of rotation diffraction data from crystals of initially unknown symmetry and cell constants.. J Appl Crystallogr.

[38] Vagin A, Teplyakov A (1997). MOLREP: an automated program for molecular replacement.. J Appl Crystallogr.

[39] Murshudov GN, Vagin AA, Dodson EJ (1997). Refinement of macromolecular structures by the maximum-likelihood method.. Acta Crystallogr D.

[40] Emsley P, Cowtan K (2004). Coot: model-building tools for molecular graphics.. Acta Crystallogr D.

[41] Kjeldsen T, Balschmidt P, Diers I, Hach M, Kaarsholm NC (2001). Expression of insulin in yeast: the importance of molecular adaptation for secretion and conversion.. Biotechnol Genet Eng.

[42] Sciacca L, Cassarino MF, Genua M, Pandini G, Le Moli R (2010). Insulin analogues differently activate insulin receptor isoforms and post-receptor signalling.. Diabetologia.

[43] Sommerfeld MR, Muller G, Tschank G, Seipke G, Habermann P (2010). *In vitro* metabolic and mitogenic signaling of insulin glargine and its metabolites.. PLoS ONE.

[44] Schäffer L (1994). A model for insulin binding to the insulin receptor.. Eur J Biochem.

[45] Wells JA (1990). Additivity of mutational effects in proteins.. Biochemistry.

[46] Kaarsholm NC, Norris K, Jørgensen RJ, Mikkelsen J, Ludvigsen S (1993). Engineering stability of the insulin monomer fold with application to structure-activity relationships.. Biochemistry.

[47] Kristensen C, Andersen AS, Østergaard S, Hansen PH, Brandt J (2002). Functional reconstitution of insulin receptor binding site from non-binding receptor fragments.. J Biol Chem.

[48] Mynarcik DC, Yu GQ, Whittaker J (1996). Alanine scanning mutagenesis of a C-terminal ligand binding domain of the insulin receptor α subunit.. J Biol Chem.

[49] Whittaker J, Whittaker L (2005). Characterization of the functional insulin binding epitopes of the full-length insulin receptor.. J Biol Chem.

[50] Whittaker J, Sørensen H, Gadsbøll VL, Hinrichsen J (2002). Comparison of the functional insulin binding epitopes of the A and B isoforms of the insulin receptor.. J Biol Chem.

[51] Kjeldsen T, Andersen AS, Wiberg FC, Rasmussen JS, Schäffer L (1991). The ligand specificities of the insulin receptor and the insulin-like growth factor-I receptor reside in different regions of a common binding site.. P Natl Acad Sci USA.

[52] Frayn KN (2010). Metabolic regulation: a human perspective.

