# Supplementary material for: Engineering of Insulin Receptor Isoform-Selective Insulin Analogues
Source: PLoS One. 2011 May 20;6(5):e20288. doi: 10.1371/journal.pone.0020288 (PMC3098868; doi:10.1371/journal.pone.0020288)
Supplement: Table S1 — Data collection and definement statistics. (DOC) [file pone.0020288.s001.doc]

**Supporting Information**

**Table S1.** **Data collection and definement statistics.**

|  | [B25H, B27E] |
| --- | --- |
| **Data collection** |  |
| Space group | I2_1_3 |
| Unit cell dimensions (Å) | a=b=c=78.9 |
| Resolution (Å) | 50 - 1.70 (1.74-1.70)* |
| No. observations | 64629 (4133) |
| No. unique reflections | 9841 (600) |
| R_merge_^§^ | 0.05 (0.53) |
| I/σ(I) | 25.9 (4.1) |
| Completeness (%) | 98.5 (99.8) |
|  |  |
| **Refinement** |  |
| Resolution range (Å) | 32–1.70 |
| No. reflections | 8598 |
| R/ R_free_^†^ | 0.19/0.22 |
| No. peptide atoms | 418 |
| No. water molecules | 84 |
| Average B-factor peptide | 10.1 |
| Water | 27.2 |
| Root mean square deviations |  |
| Bond lengths (Å) | 0.03 |
| Bond angles (^o^) | 2.0 |
| Ramachandran |  |
| Residues in most favoured regions (%) | 97.7 |
| Residues in allowed regions (%) | 2.3 |

^*^ Values in parenthesis are for the highest resolution shell

^§^Rmerge = Σ|Ii - I|/ Σ I where Ii is an individual intensity measurement and I is the mean intensity for this reflection.

^†^ R = crystallographic R-factor = Σ |F_obs_| - |F_calc_|/ Σ |F_obs_|, where F_obs_ and F_calc_ are the observed and calculated structure factors respectively. R_free_ value is the same as R value, but calculated on 5% of the data not included in the refinement.
